# Caution is warranted when using animal space-use and movement to infer behavioral states

**DOI:** 10.1186/s40462-021-00264-8

**Published:** 2021-06-11

**Authors:** Frances E. Buderman, Tess M. Gingery, Duane R. Diefenbach, Laura C. Gigliotti, Danielle Begley-Miller, Marc M. McDill, Bret D. Wallingford, Christopher S. Rosenberry, Patrick J. Drohan

**Affiliations:** 1 Department of Ecosystem Science and Management, Pennsylvania State University, University Park, PA 16802 USA; 2 Pennsylvania Cooperative Fish and Wildlife Research Unit, Pennsylvania State University, University Park, PA 16802 USA; 3 U. S. Geological Survey, Pennsylvania Cooperative Fish and Wildlife Research Unit, Pennsylvania State University, University Park, PA 16802 USA; 4 Department of Environmental Science, Policy, and Management, University of California Berkeley, Berkeley, CA 94720 USA; 5 Teatown Lake Reservation, Ossining, NY 10562 USA; 6 Pennsylvania Game Commission, Harrisburg, PA 17110 USA

**Keywords:** Breeding, Mate search strategy, *Odocoileus virginianus*, White-tailed deer, Hidden Markov models, Home-range, Utilization distribution, Brownian bridge, Behavioral state, State identification

## Abstract

**Background:**

Identifying the behavioral state for wild animals that can’t be directly observed is of growing interest to the ecological community. Advances in telemetry technology and statistical methodologies allow researchers to use space-use and movement metrics to infer the underlying, latent, behavioral state of an animal without direct observations. For example, researchers studying ungulate ecology have started using these methods to quantify behaviors related to mating strategies. However, little work has been done to determine if assumed behaviors inferred from movement and space-use patterns correspond to actual behaviors of individuals.

**Methods:**

Using a dataset with male and female white-tailed deer location data, we evaluated the ability of these two methods to correctly identify male-female interaction events (MFIEs). We identified MFIEs using the proximity of their locations in space as indicators of when mating could have occurred. We then tested the ability of utilization distributions (UDs) and hidden Markov models (HMMs) rendered with single sex location data to identify these events.

**Results:**

For white-tailed deer, male and female space-use and movement behavior did not vary consistently when with a potential mate. There was no evidence that a probability contour threshold based on UD volume applied to an individual’s UD could be used to identify MFIEs. Additionally, HMMs were unable to identify MFIEs, as single MFIEs were often split across multiple states and the primary state of each MFIE was not consistent across events.

**Conclusions:**

Caution is warranted when interpreting behavioral insights rendered from statistical models applied to location data, particularly when there is no form of validation data. For these models to detect latent behaviors, the individual needs to exhibit a consistently different type of space-use and movement when engaged in the behavior. Unvalidated assumptions about that relationship may lead to incorrect inference about mating strategies or other behaviors.

**Supplementary Information:**

The online version contains supplementary material available at 10.1186/s40462-021-00264-8.

## Background

Animal movement is a critical component of many individual- and population-level processes, such as space-use (e.g., [46]), gene flow (e.g., [14]), disease dynamics (e.g., [28]), range expansion (e.g., [7]), and population dynamics (e.g., [51]). Inference on animal movement is typically obtained by monitoring time-indexed locations from animal-borne sensors, and our ability to obtain precise estimates of these locations over a long period of time and at a fine-temporal scale has become logistically easier and more cost effective over time [13, 58, 62, 66]. Along with a proliferation of datasets containing positional data on individual animals, researchers have developed a wide variety of tools and statistical models to visualize, quantify, and predict animal movement and space-use [37]. Some of these methods focus on a specific aspect of movement ecology, which is the identification of the underlying, latent, behavioral state of an individual that results in variation in movement and space-use quantities [29]. Behavioral state identification allows researchers to estimate when an individual was engaged in a behavior (e.g., resting, foraging, exploring, transiting, excursions, dispersal) and variables that may contribute to the display of one behavior over another (e.g., [17, 23, 48, 49, 57, 67]).

Given the proliferation of methods for behavioral state identification, we foresee researchers using these methods to identify increasingly complex behavioral states that are important for understanding an animal’s ecology. For example, mating-related movement strategies are an important component of an individual’s lifetime reproductive success and fitness [24], although studies that link fitness directly to movement strategies are rare [52]. Knowing the timing and location of mating events can indicate which search strategies are successful and provides insight into the fitness trade-offs necessary for reproductive success [26]. However, classifying behavioral states using location data is difficult without supporting data about the resources (e.g., mates) that are related to movement behavior [33]. In addition, previous research has found that the current suite of methods for identifying latent behavioral states may not match the true behavioral state [5]. The combination of rich location-based datasets, accessible but complex statistical methods, and the absence of supporting data for validation purposes can create the perfect storm for a mismatch between the desired inference and the limitations of the data and statistical model.

White-tailed deer are a model species to evaluate methods for characterizing mating-related movement strategies because they are highly mobile and physically large enough to support global positioning system (GPS) collars that monitor movement over long periods of time and at a fine temporal scale. As scramble competitors, physical traits of males are weakly correlated with reproductive success [18, 36], suggesting mate search efforts are critical to successful reproduction and fitness [40]. Several search strategies have been identified for each sex using an individual’s search intensity (size or proportion of home range used during the mating season) and movement rate (m/hr) (e.g., [35, 60, 65]). Low movement rates and small home ranges suggest a female sit-and-wait strategy, which is in contrast to the female excursion strategy, a potential form of female mate choice, where movement rates and home range sizes are greater as females occasionally travel outside the home range [16, 39, 60]. As scramble competitors, male white-tailed deer movement strategies are influenced by the number and behavior of competing males and the probability of encountering a female [35, 65]. When there is a low density of females and travel time between females is high, males should reduce their movement rates and focus their activity in the portions of their home range that are in close proximity to females (resident strategy; [26, 65]). When travel time between females is low males may increase movements to increase encounters with females (roving strategy; [65]).

At the discovery of a potential mate, male white-tailed deer may engage in multiple behaviors to increase their reproductive success. White-tailed deer form tending bonds where the male isolates with the female until the end of estrus, during which time mating likely occurs (tending; [1, 2, 32]), although the male may be displaced by another male. However, our knowledge of the behavior exhibited during tending bonds is limited to studies on captive deer [19]. In systems where estrus is not synchronized and receptivity is difficult to predict, it may be advantageous for males to engage in a roving strategy [65]; in white-tailed deer it has been hypothesized that males may engage in roving behavior but revisit known females to check their estrus status during rut (revisitation; [25]). Given the difficulty of making field observations of mating-related behavior, there is interest in using location-only data to infer behavior related to mating strategies (e.g., sit-and-wait/resident, excursion, tending, revisitation).

Due to logistical constraints, inference on mating movement behavior is often limited to data from a single sex and/or the occurrence of a mating event is unknown [59]. For example, revisited focal areas (identified using the 30% probability contour from a utilization distribution) from male-only location data have been hypothesized to reflect areas that contain potentially receptive females [25]. These males may be mirroring the space-use of resident females, whose home-ranges are typically smaller than males, particularly during the mating season [4, 34], but it is unknown if males interacted with females in those focal areas. From female-only location data, female excursions outside of the home range (identified using the 95% isopleth from a utilization distribution) were inferred to increase encounters with males and mating success [39], which can be validated with estimates of conception date (e.g., [60]). When information is only obtained on a single sex, however, hypotheses about mate interactions rely on strong assumptions about how space-use and movement characteristics represent mating behavior. We believe these strong assumptions need to be validated before we can infer mating behavior from movement characteristics of individuals alone.

Using fine-scale, concurrent location data from both sexes, we evaluated whether two commonly used movement analyses, utilization distributions (UDs) and hidden Markov models (HMMs), could be used to differentiate mate-interaction from non-mate-interaction behavior in white-tailed deer. We were motivated to evaluate the ability of UDs to detect mate-interaction behavior because they have been used previously as a method for defining mate-search behavior using single-sex location (e.g., [26, 39, 60]). While we have not seen HMMs used explicitly for the purpose of identifying mate-interaction behavior, they are increasingly being used by ecologists to determine behavioral movement states, which is likely due to their accessibility to ecologists through R packages such as moveHMM [50] and momentuHMM [47]. For each method, we used the location of male-female interaction events (MFIEs) to determine if the observed interactions fell within areas of consistent UD volumes or within a consistent behavioral state. Consistency of either method to detect MFIEs would be evidence that space-use or movement varied in a predictable way between times when males or females were with or without a potential mate.

## Methods

### Study area

We monitored deer in four study areas in Pennsylvania, United States, where seasons were characterized by cold winters (mean temperature − 4.4 °C**)** and humid summers (mean temperature 20.5 °C) [54]. Two study areas, Susquehannock North (SN) and Susquehannock South (SS), were located in Susquehannock State Forest in Potter County. Our study area was contained within the Appalachian Plateau physiographic region with plateaus at approximately 800 m elevation interspersed with drainages dropping to 220 m [15]. The area was predominately forested and dominant tree species were red maple (*Acer rubrum*), sugar maple (*Acer saccharum*), black cherry (*Prunus serotina*), and American beech (*Fagus grandifolia*) [3]. Two additional study areas were in Rothrock (RR) and Bald Eagle (BE) state forests. Located in Centre, Mifflin, and Huntingdon counties, RR and BE were within the Ridge and Valley Physiographic province with topographic features that consisted of long, parallel ridges and valleys along a northeast-southwest axis, elevation ranging from 400 to 700 m above sea level [15]. Predominately forested, dominant tree species were red and white oak (*Quercus* spp.), red maple, black birch (*Betula lenta*), black gum (*Nyssa sylvatica*) and hickory (*Carya* spp.). The oak-hickory forests contained an understory layer of ericaceous shrub species (*Vaccinium* spp., *Gaylussacia* spp., and *Kalmia latifolia*). White-tailed deer density on our study areas ranged from 4 to 10 deer/km^2^ [64].

### Animal capture and data collection

We captured deer using rocket nets and Clover traps from January to April, 2013–2016 [30]. We followed protocols approved by The Pennsylvania State University Institutional and Animal Care and Use Committee (Protocol No. 47054). We fitted deer with a GPS satellite collar (GPS Plus, Vectronic Aerospace, Berlin, Germany) programmed to obtain a location every 1 h. All deer in this study were ≥ 2.5 years of age.

### Data analyses

#### Identifying male-female interaction events

We identified MFIEs using GPS locations from male and female deer within the same study area. During the rut, male deer tend a female for up to 72 h, during which time mating likely occurs [19]. It is also possible that a male will find a female (or group of females) that has not entered estrus and, instead of tending, will revisit areas where the female was located assess her receptivity [25]. Therefore, times when male and female deer are in close proximity to one another can indicate probable male-female interaction events. We also defined three breeding phases, during which time individuals may exhibit different behavior within the annual breeding season. For the northern study area, the breeding phases were defined as: October 18–November 7 (early), November 8–November 28 (peak), and November 29–December 19 (late; [21]). For the southern study area, the breeding phases were defined as: October 16–November 5 (early), November 6–November 29 (peak), and November 30–December 20 (late [21];). Analyzing phases separately may provide us with additional insight into the ability of the two methods to detect potential breeding events, however, there are also fewer locations within each phase on which to make inference. In Pennsylvania, the early and peak rut seasons occur prior to the start of rifle season but during archery season; however, hunter density is low during archery season compared to rifle season. In addition, harvest regulation changes intended to increase the number of older males in the population did not result in changes to timing of breeding or female productivity (embryos/female; [21]).

We used both a liberal and conservative approach to identifying MFIEs. There is little information on how white-tailed deer move during a potential mate-interaction because few studies monitor both males and females; using both classification methods allowed us to capture potentially different mate-interaction behavior. For the liberal approach, we used the Prox function within the WildlifeDI R package [42] to calculate the distance between male-female pairs based on simultaneous locations. We defined MFIEs as events when a male and female deer were within an average of 100 m of each other for a minimum of 2 continuous locations (one hour). We are not aware of any data on the distance between males and females during tending or the distance necessary for a male to assess estrus state of a female. If the larger distances were non-mate-interactions and the analytic method performed well, we would see this in the results (i.e., at short distances the method worked well but not at large distances). For individual pairs with repeated interactions, we delineated mating events based on more than 100 m between the individuals for longer than a two-hour period. Repeated interactions may indicate revisitation behavior, as opposed to tending; however, we classify both as mate-interaction behavior.

**Fig. 1:**
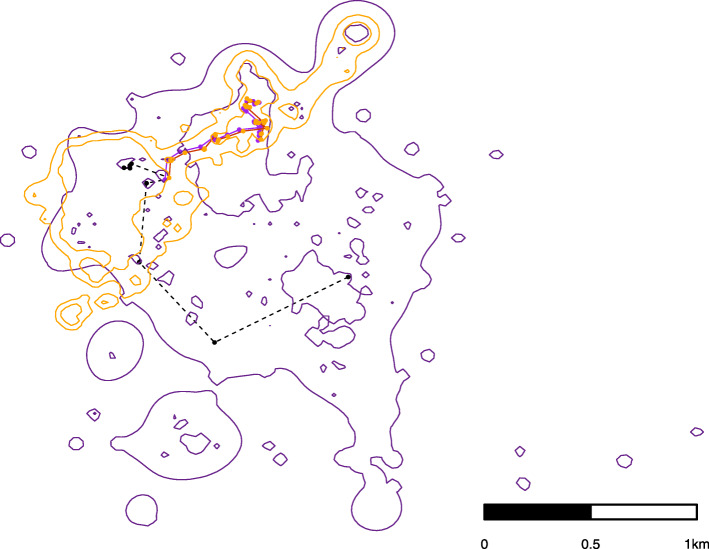
Example male-female interaction events (MFIE) where black points are pre-event locations and orange and purple points represent female and male locations, respectively, during the MFIE. The contours correspond to 95 and 50% probability contours of the utilization distribution (UD)

For the conservative approach, we identified MFIEs using a combination of proximity and similarity of movement trajectories. To determine similarity of movement trajectories, we used the DI function within the WildlifeDI R package [42] to calculate dynamic interaction (DI) statistics for male-female pairs. This metric compares the similarity of two animals’ movement trajectories in relation to movement displacement (direction and speed), where values near − 1 indicate opposing movement displacement, values near 0 indicate random movement, and values near 1 indicate cohesive movement displacement [42, 43]. To obtain a conservative estimate of MFIEs, we calculated the DI for each segment of movement trajectory associated with the MFIEs identified by the liberal method and retained those events with an average DI of greater than 0.5. A DI value of greater than 0.5 indicates that two deer are moving similarly within regards to their direction and speed of movement [43]. Therefore, the conservative set of MFIEs were events when a male and female deer were within 100 m, on average, of each other, for a minimum of 2 continuous locations and exhibited cohesive movement. An example of a MFIE can be found in Fig. 1.

#### Evaluating the UD approach for identifying MFIEs

The Brownian bridge movement model (BBMM) is a method for quantifying an individual’s UD, or the relative frequency of use across a given period of time, that accounts for temporal autocorrelation in sequential observations and measurement error. Many methods exist to fit a BBMM to data, and we present one method which can be implemented in R using existing packages. Previous studies have inferred mating-related behavior from male-only data by hypothesizing that locations that occurred within an a priori probability contour represented locations of potentially receptive breeding females (e.g., 30% in [25]), and from female-only data, when females travelled outside of an a priori probability contour (e.g., 95% in [39, 60]). Therefore, we assessed the UD approach for both males and females and based inference on the standardized UD volume of each location in a MFIE. The standardized UD volumes are used to construct probability contours by starting at the areas of highest intensity use and then sequentially including areas of less intensely used areas until the cumulative volume reaches some desired proportion of the total volume; for example, a grid-cell with a UD volume of 30% would be contained within probability contours greater than or equal to 30% but not in those less than 30%. Typically, the 95% probability contour is used to define a home-range of an animal [22].

**Fig. 2:**
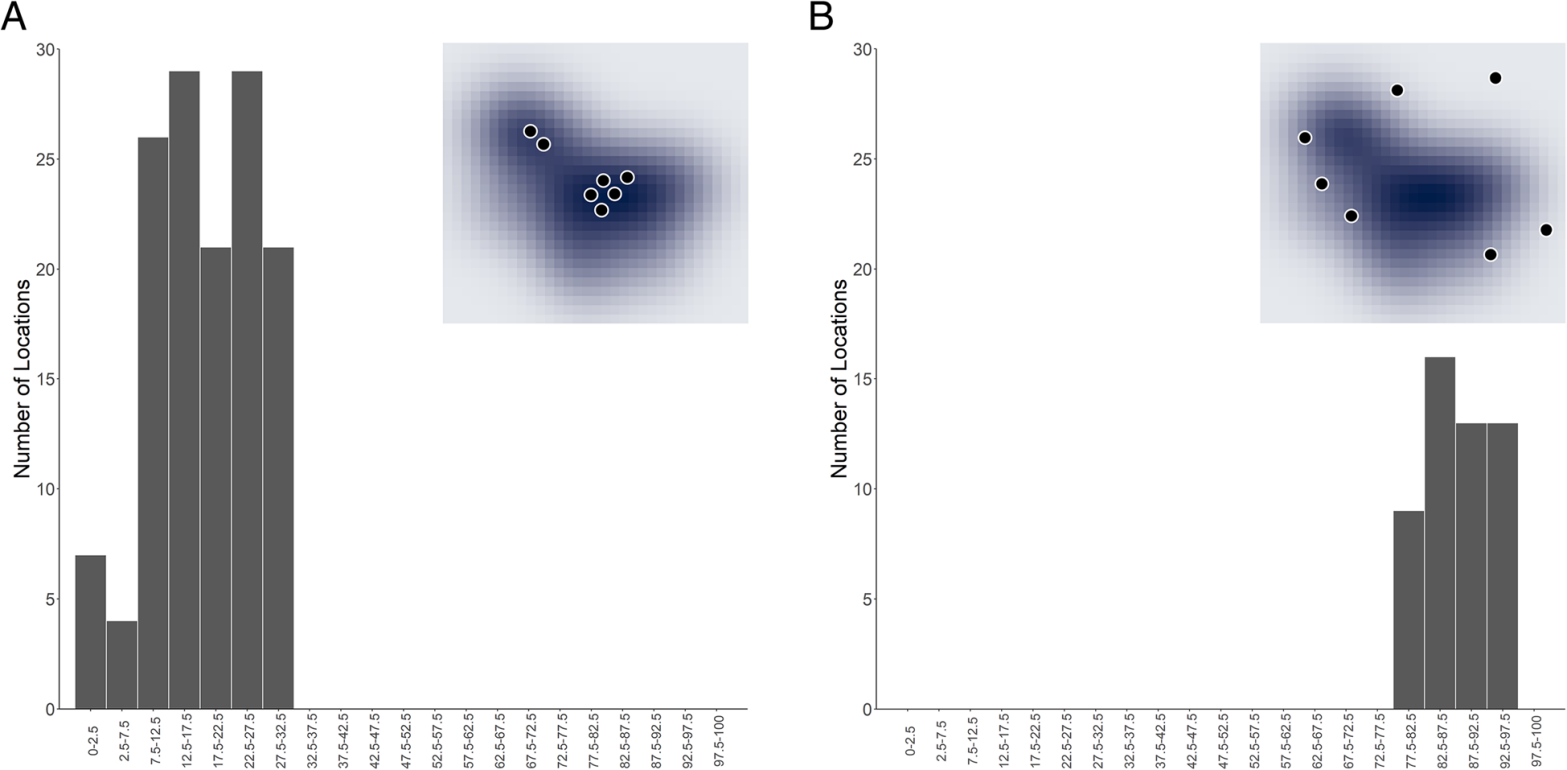
Expected results from the utilization distribution (UD) approach if it was able to consistently identify male-female interaction events (MFIEs). Subfigure A demonstrates the scenario in which MFIEs were in high-use areas (small UD volumes) and subfigure B is the scenario in which MFIEs were in low-use areas or outside of core areas (large UD volumes). The inset shows an example UD with locations in high-use (**A**) and low-use areas (**B**)

We used the BBMM package [55] and the function brownian.bridge to estimate the parameters of the BBMM. We fixed the measurement error to a standard deviation of 10 m, based on field testing of the GPS collars, and estimated the BBMM over a regular grid with a spatial resolution of 30 m. We fit the BBMM to each individual separately, in two ways; we fit a single BBMM to all locations within an annual breeding season, and we fit a single BBMM to all locations within a breeding phase (early, peak, and late). We then determined the UD volume of each grid-cell, using the function getvolumeUD in the adehabitatHR package [11], that contained the coordinates of locations identified as MFIEs. If the MFIE locations had a UD volume that is consistently less than some value for males (e.g., [25]) or greater than some value for females (e.g., [39, 60]), then we can say that the UD approach can determine mate-interaction events from single-sex location data. See Fig. 2 for expected results if MFIE locations were consistently found within a particular range of UD volumes.

#### Evaluating HMM for identifying MFIEs from single sex data

**Fig. 3:**
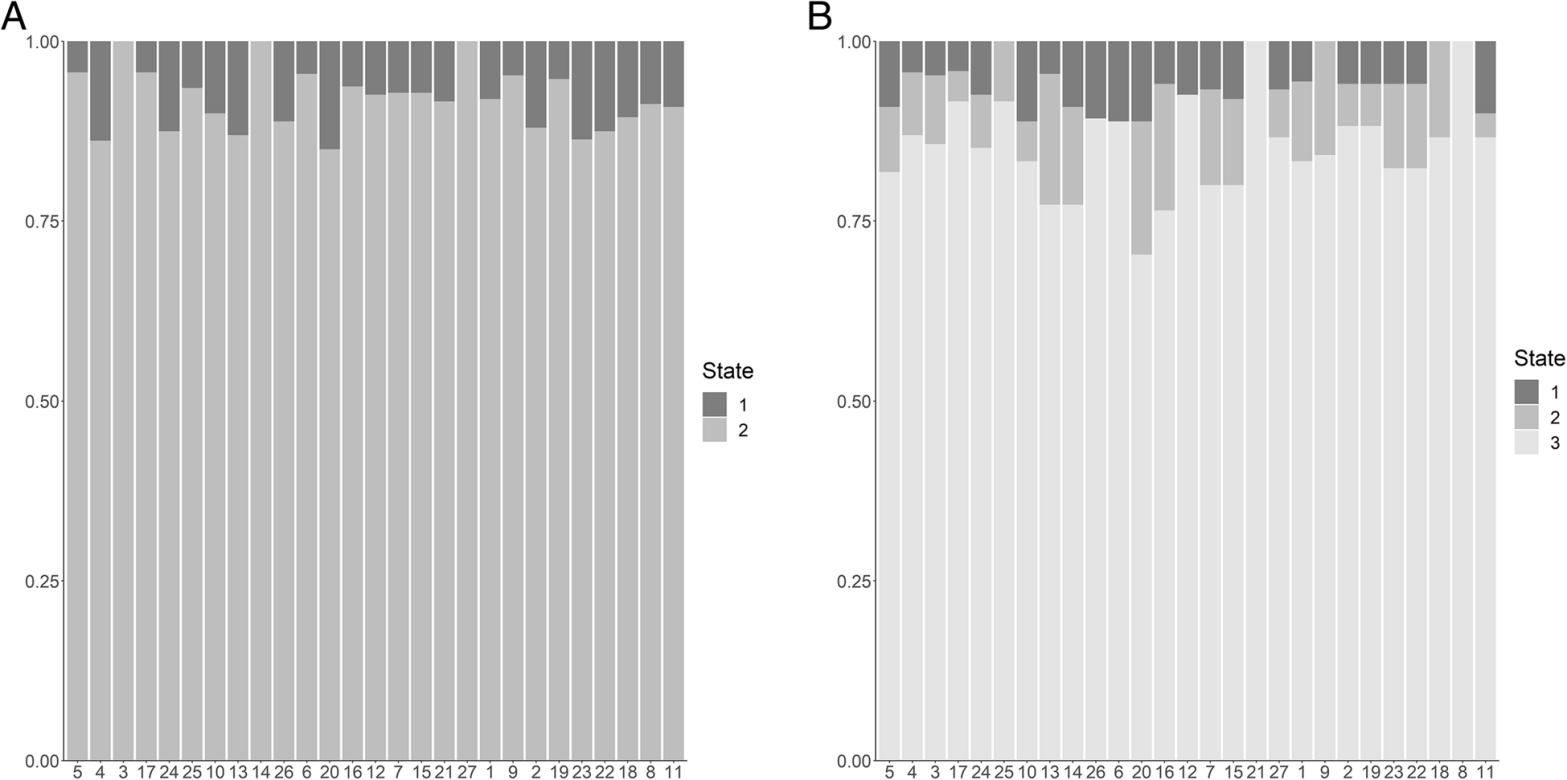
Expected results from the hidden Markov model (HMM) approach if it was able to consistently identify male-female interaction events (MFIEs) for a 2-state (**A**) and a 3-state model (**B**). Ideally all locations in each event would be identified as a single, consistent state across events (the actual state label does not matter), but we would also expect that there may be some small proportion of locations within each event classified as another state

Hidden Markov Models (HMMs) have become a popular movement model for describing movement characteristics (speed and turning angle) that are assumed to arise from different behavioral states. To fit a HMM, we first needed to obtain locations at regular intervals. Therefore, we used the fmove.bayes function from the ctmcmove package [31] to fit a continuous-time functional movement model [10] with a fixed measurement error (SD = 10 m) and a CAR1 process error covariance matrix. We used this model to estimate locations on an hourly interval using 10,000 MCMC iterations; the posterior mean of each location was used as the data in the subsequent HMM analysis. We used the momentuHMM package [47] to fit the HMMs and process the output. Similar to the UD approach, we fit HMMs to both the full set of breeding season movement paths and to breeding-phase specific movement paths (the subset of hourly locations that fell into each breeding phase). We also fit both two- and three-state HMMs; selecting the appropriate number of states for an HMM is notoriously difficult as many model selection methods overestimate the number of states [56]. We hypothesized that deer movement during the rut could be characterized by two states or by three states. The addition of a third state would allow for more flexibility in categorizing the movement trajectory (e.g., foraging, resting, mate-interaction as opposed to a subset of two behaviors). The HMM does not tell you what behavior the state corresponds to; interpretation of the biological meaning of the resulting states and their parameters is up to the researcher. The HMMs were fit jointly, allowing the state parameters to be shared among individuals; however, this method does assume that movement quantities during the states of interest arise from the same population-level distribution, which allows for little individual variation. Because data used in the HMM model is predicted hourly, we used the start and end times of the MFIEs to denote MFIE locations in the continuous-time framework (such that all hourly locations between the start and end times of an MFIE were considered MFIE locations). The momentuHMM package calculates the most likely state sequence using the Viterbi algorithm. If the MFIE locations were consistently categorized as belonging to one state over another, then we could say that the HMM can determine mate-interaction events from single-sex location data. In Fig. 3 we show our expected results if the HMM approach was successful at identifying MFIE.

## Results

Using the liberal filter, we identified 27 MFIEs across 5 males and 7 females where MFIEs averaged 7.9 h (minimum = 1 h, maximum = 78.9 h). Using the conservative filter, we identified 18 MFIEs across 4 males and 5 females where MFIEs averaged 7.1 h (min = 1 h, max = 27 h). A summary of each MFIE is in Additional file 1 (Table A1–1 and A1–2). There was no evidence that either a UD volume threshold a HMM based on single-sex location data was able to identify MFIEs.

**Fig. 4:**
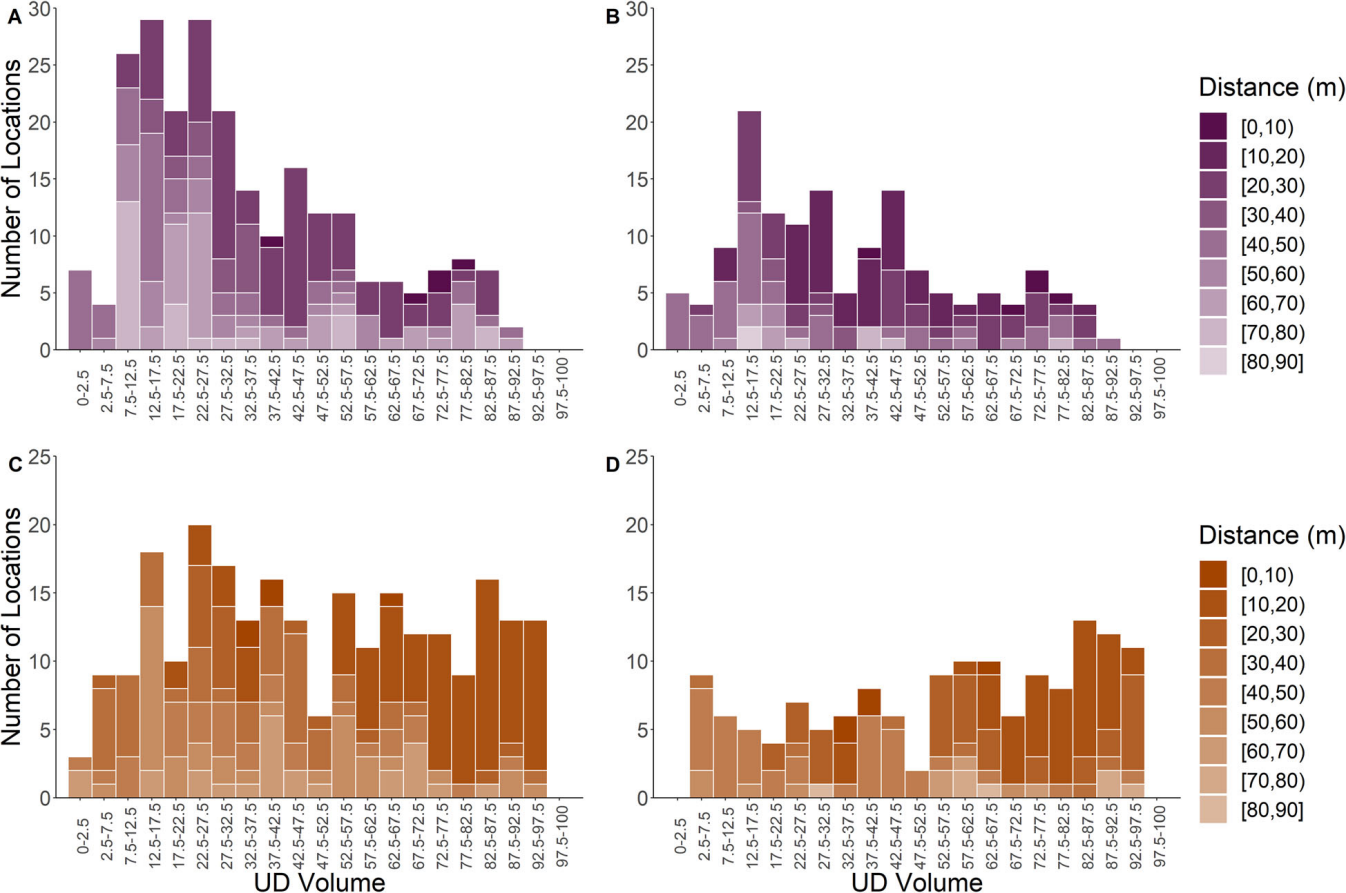
Histogram of the UD volume (the smallest probability density contour that would contain the location) associated with white-tailed deer locations occurring during a male-female interaction event (MFIE) within the annual breeding season. MFIEs were identified using a liberal and conservative identification method for males (**A** and **B** respectively) and females (**C** and **D** respectively). The UD volume for each point was calculated as arising from a utilization distribution over the annual breeding season. The shading represents the average distance between a male and female during the MFIE associated with each location

**Fig. 5:**
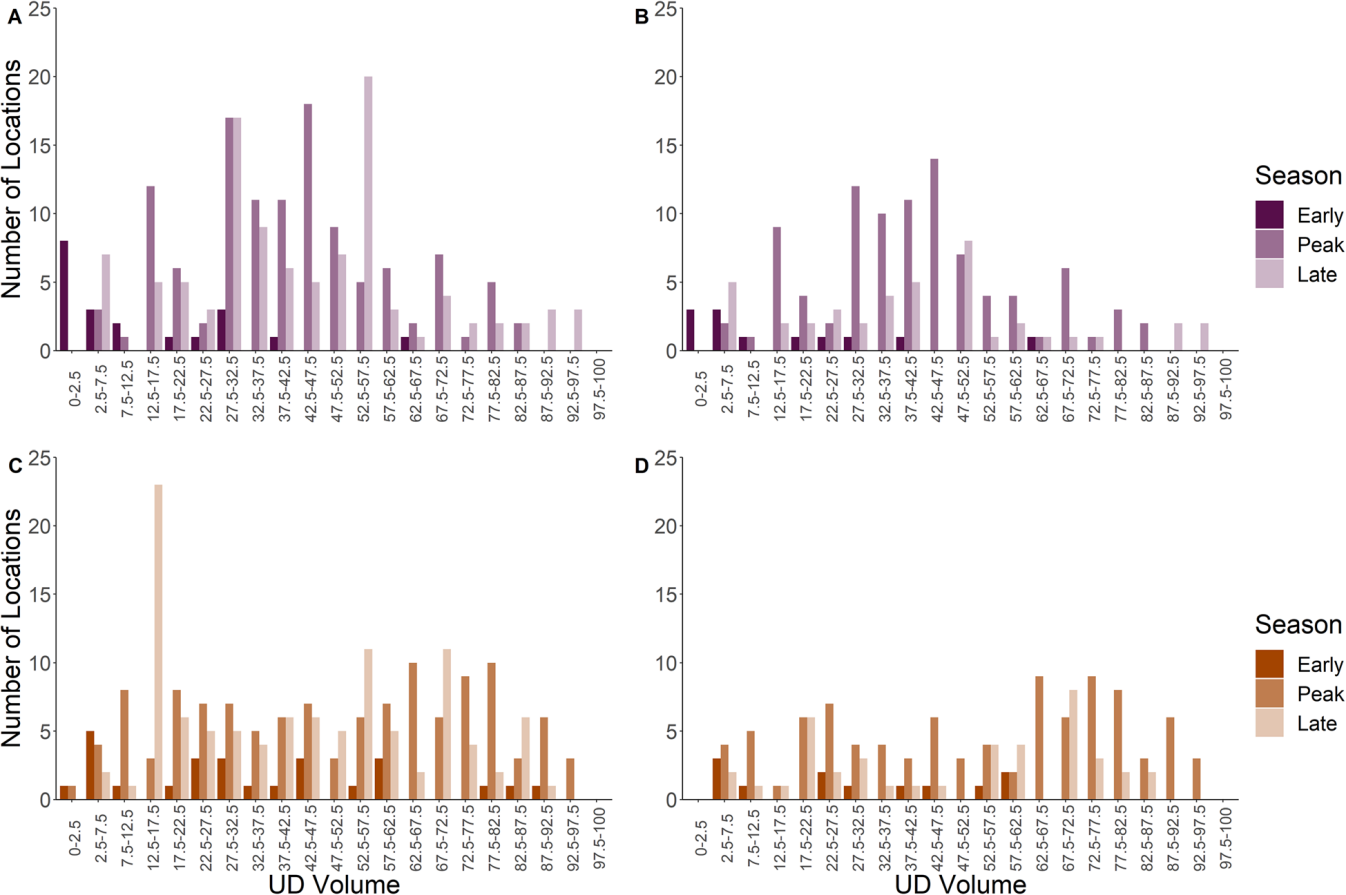
Histogram of the UD volume associated with white-tailed deer locations occurring during a male-female interaction event for early, peak, and late phases of the annual breeding season. Male-female interaction events were identified using a liberal and conservative identification method for males (**A** and **B** respectively) and females (**C** and **D** respectively). The UD volume for each point was calculated as arising from a utilization distribution over each phase within the breeding season

For locations identified as occurring during an MFIE, the standardized UD volume spanned 2–91% for males (Fig. 4A and B) and 1–97% for females (Fig. 4C and D). For males, the average UD for each MFIE (the UD values for the cells traversed by an individual during a MFIE) ranged from 9.46–75.80% and 10.98–75.80% using the liberal and conservative MFIE classification methods, respectively (Table A1–3 and A1–4). For females, the average UD for each MFIE ranged from 1.76–75.66% and 14.22–84.04% using the liberal and conservative MFIE classification methods, respectively (Table A1–3 and A1–4). We did not see a relationship between the performance of the method and either the average distance between a male and a female during a MFIE (Fig. 4) or the duration of the MFIE (Fig. A2–1 in Additional file 2). We also failed to detect a seasonal difference for either sex in the ability of the UD to delineate MFIE in different breeding phases (Fig. 5).

**Fig. 6:**
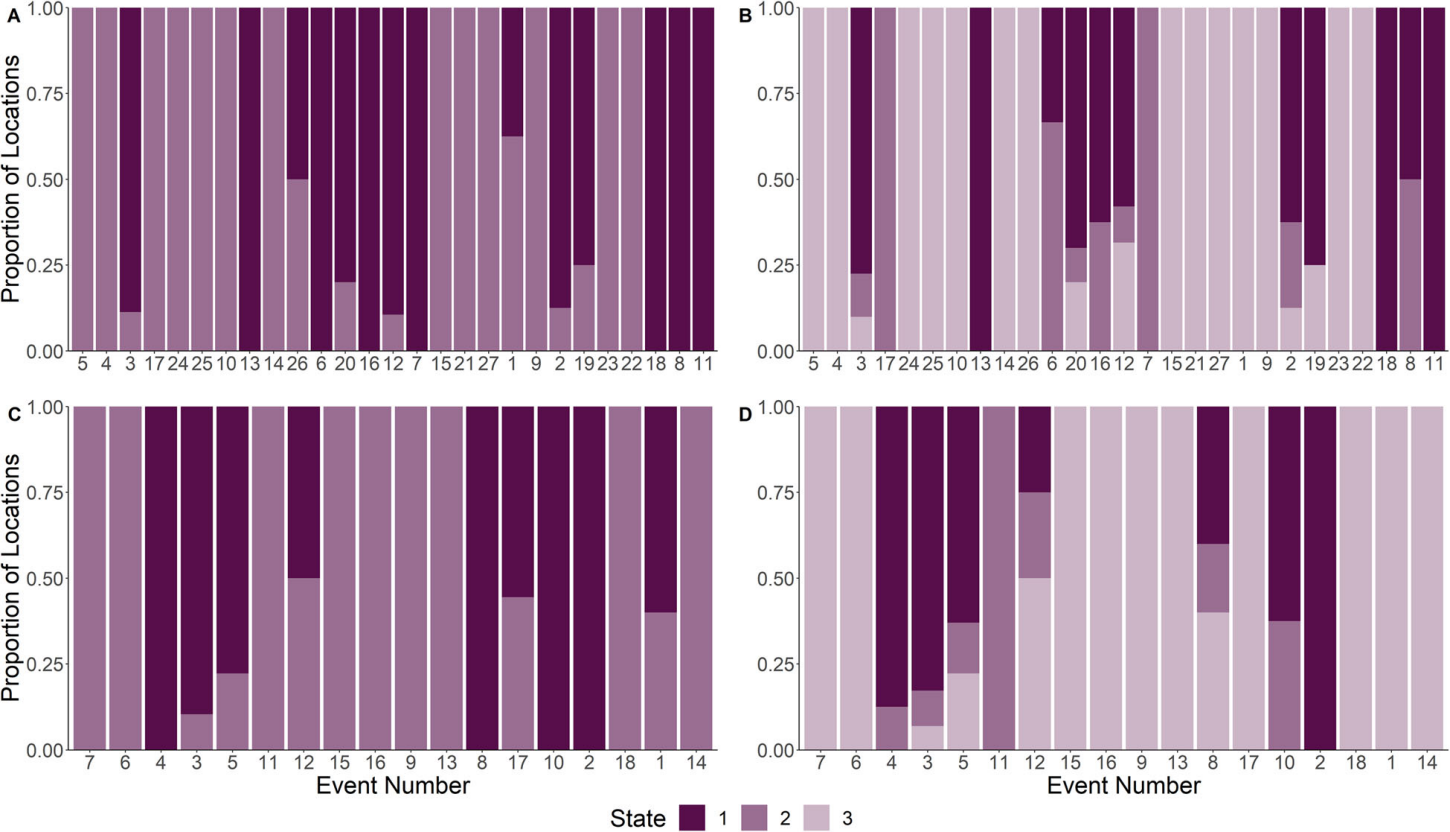
Proportions of male white-tailed deer locations identified as arising from two- (**A** and **C**) and three- state (**B** and **D**) HMMs during male-female interaction events (MFIEs) that were classified using the liberal event identification method (**A** and **B** respectively) and the conservative event identification method (**C** and **D** respectively) where HMMs were fit to telemetry data across the breeding season. Events are ordered from small to large average distance between a male and female during the MFIE

Using male-only data, the HMM was unable to identify the MFIEs (Fig. 3). To visualize the results, we calculated the proportion of locations within each MFIE that were categorized as belonging to each state. Single events were often split across multiple states, and the predominant state that each event was categorized as was not consistent across events (Fig. 6 and A2–2). The classification ability of the HMM did not improve when restricted to single breeding phases within a breeding season (Fig. A2–3, A2–4, A2–5, and A2–6). There was also no relationship between the performance of the method and either the average distance between a male and a female during a MFIE (Fig. 6, A2–3, and A2–5) or the duration of the MFIE (Fig. A2–2, A2–4, and A2–6).

## Discussion

Variation in movement metrics while in the presence of an individual of the opposite sex was large both within and among individuals; therefore, it is difficult for statistical models to infer from single-sex location data where and when an individual was engaged in a MFIE. Regardless of MFIE classification scheme, sex, or breeding season, there was no evidence of a UD volume threshold for locations identified as an MFIE. We observed that some UD volumes contained more MFIEs locations than others, which could provide some support that UDs may reflect mate interactions [25]; however, MFIEs were not consistently restricted to a narrow range of UD volumes and assuming MFIEs only occurred in these volumes would ignore a significant portion of MFIEs. Defining a MFIE as having occurred based on a UD volume threshold alone will likely incorrectly identify the number and location of potential mate interactions. Therefore, researchers should be cautious when interpreting events that have been identified based solely on single-sex location data. For example, previous studies using male-only location data made inference about mate-searching behavior and assumed that areas of high use by males represented the locations of females [25]. However, with no data on females, focal areas identified in a UD analysis could alternatively contain a resource that is important for deer survival (e.g., cover, water, forage). During MFIEs, male movement patterns were not consistently restricted to a small probability contour (e.g., < 30%) which may be because females did not remain stationary throughout the rut. Although some males may exhibit stationary movement behaviors during a breeding season, labeling such movements as corresponding to an interaction with a potential mate could not be supported by our analyses.

Presumed female mate-interaction events also were not detected using female-only location data and a UD approach. Females used portions of their home range during MFIEs that were associated with varying UD volume. In addition to individual variation, female mating strategies (sit-and-wait or excursions) are hypothesized to depend on population densities and sex ratios [34]. When only female locations are available, using auxiliary information, such as back-calculating the conception date (e.g., [60]), is recommended to identify the mating location and its relationship to the individual’s typical space-use.

Hidden Markov models also were incapable of classifying movements in the presence of the opposite sex as a unique behavioral state. HMMs identify behavioral states by detecting a change in movement rate and direction (i.e., turning angle). Therefore, the inability of HMMs to correctly classify MFIE suggests that movement behavior during mate-interaction is not consistently different from other behavioral states (i.e., searching). As such, it is likely difficult to determine when an individual shifts their behavior from mate searching to mate interaction using location data alone. It is tempting to think that HMMs can directly estimate behavioral modes; however, the HMM is estimating parameters of distributions, the number of which is specified a priori by the researcher, that give rise to the observed step lengths and turning angles. It is the researcher who ascribes a behavioral interpretation to the estimated distribution. If the behavior does not result in a consistent and interpretable partitioning of the movement path, then the HMM will not allow the researcher to make inference on the unobserved behavioral state. In addition, if individuals are mirroring another individual’s movement behavior during the behavior of interest (e.g., attempted mating; [2]), then single-sex location data would not be consistently identified as a distinct state even though the behavior is occurring.

The primary mortality risk factor for white-tailed deer in our study area is hunting-induced mortality, which varies in intensity in both time and space and across sex- and age-classes. The three rut periods analyzed in our study differ in the degree of hunting pressure applied to the deer population, because the late rut-period overlaps with the rifle season and greater hunter densities. However, we did not detect any change in the ability of either method to identify MFIE across the rut periods. Deer have been observed adjusting their movement behavior during hunting seasons (e.g., [38, 41, 61]), which could potentially alter the ability of these methods to detect male-female interaction events. Integral to the ability of both methods to identify MFIE is that the behavior or space-use needs to be different than what the individual exhibits when not engaged in a MFIE during the temporal period in question; different movement strategies related to hunter avoidance could increase or decrease the ability of these models to identify MFIE.

We did not find support for using single-sex location data and UDs or HMMs to identify when an individual is with a potential mate. To identify mating events using fine-scale movement data, auxiliary data about the underlying behavioral states (e.g., conception date, in situ observations) or location of available resources (e.g. a mate) are needed. Increasing the sampling frequency of observations would be most beneficial when both males and females are collared, because it would allow for greater certainty that a MFIE occurred. These events could be used to provide direct inference on a MFIE, as locations of a known behavioral state in a hidden Markov model, or they could be used in a supervised machine learning framework given a sufficient number of events (e.g., [9, 12, 27, 45, 53]). Animal-borne video collars are also a promising avenue by which to obtain direct observations of the previously unobservable behavior (e.g., [6, 44, 63]). More generally, if the goal is to identify an unobserved behavioral state of interest, increasing the sampling frequency of individual locations would be ineffective if space-use and movement quantities truly don’t vary with the state of interest. Although our study focused on identifying mating behavior in white-tailed deer, the results are relevant for any study attempting to identify unobserved behavioral states from space-use and movement data.

## Conclusions

Incorrectly assigning behaviors to movement patterns is likely to lead to an incorrect understanding of the trade-offs associated with mating strategies. For example, an explanatory hypothesis for drivers of space-use based on single-sex location data may misidentify the trade-offs an individual makes to visit such locations if mate interactions do not actually occur in those areas. Instead, movements ascribed to interactions with potential mates simply may reflect trade-offs made to acquire other resources or reduce predation risk. In addition, some methods assume that mating resources (e.g., females) are relatively stationary, which may fail to detect tending, revisitation, or mating events between mobile individuals. Models that incorporate independent data, such as conception data [60], resource availability [33], molecular evaluations [20, 26], or in situ observations (via field observations or video camera collars [8];), will reduce the need for assumptions about underlying behavioral states that are currently inferred from location data.

Our study demonstrates that although UDs and HMMs may be readily accessible statistical methods for ecologists to use to identify behavior, we did not find that they were able to consistently identify male-female interactions among white-tailed deer during the breeding season. Our work highlights the importance of verifying the primary underlying assumptions made when using these methods to identify any behavior, which is that space-use and movement differ in a consistent way during the behavior of interest and differences are related to that behavior. For example, male white-tailed deer engage in scramble-competition for mates, and this strategy may produce too much variation in space-use and movement during mate-interactions to be identifiable using single-sex location data and UDs or HMMs. Differences in space-use and movement may be further modified by individual variation landscape-level processes, such as concentration of resources and hunting intensity, which only increases the need to incorporate validation data if the objective is to identify behavioral states. Therefore, ecologists should use caution when interpreting spatial and movement patterns observed in UDs and HMMs as being indicators of specific behavioral modes and suggest that auxiliary data are necessary to validate the behavioral-inference obtained from these two methods.

## Supplementary Information

**Additional file 1.** Summaries of MFIEs using liberal and conservative methods for identification and average UD volumes for each MFIE.

**Additional file 2.** PDF including a visual representation of an example MFIE, expected results for the UD and HMM approach, and a summary of each male-female interaction event.

## Data Availability

The datset supporting the conclusions of this article is archived in the Dryad repository (10.5061/dryad.mgqnk98zz).

## Abbreviations

BBMM: Brownian bridge movement model
MFIE: Male-female interaction event
HMM: Hidden Markov model
UD: Utilization distribution
