# Supplementary material for: Caution is warranted when using animal space-use and movement to infer behavioral states
Source: Mov Ecol. 2021 Jun 11;9:30. doi: 10.1186/s40462-021-00264-8 (PMC8196457; doi:10.1186/s40462-021-00264-8)
Supplement: Supplementary file 1 — Additional file 1. Summaries of MFIEs using liberal and conservative methods for identification and average UD volumes for each MFIE. [file 40462_2021_264_MOESM1_ESM.docx]

**Additional File 1**

Table A1-1: Summary of male-female interaction events (MFIE) using the liberal method for event identification.

| Male ID | Female ID | Start | End | Duration (h) | Mean Distance (m) | Mean DI |
| --- | --- | --- | --- | --- | --- | --- |
| 13686_13732 | 8101_8102 | 11/11/16 00:01:14 | 11/11/16 07:00:42 | 6.99 | 63.93 | 0.29 |
| 8111_8112 | 8101_8102 | 11/07/16 10:00:11 | 11/07/16 17:00:10 | 7.00 | 66.94 | 0.13 |
| 8111_8112 | 8101_8102 | 11/08/16 01:00:58 | 11/11/16 08:00:09 | 78.99 | 21.21 | 0.47 |
| 8111_8112 | 8101_8102 | 11/04/14 09:00:43 | 11/04/14 12:00:38 | 3.00 | 9.59 | 0.57 |
| 8111_8112 | 8101_8102 | 11/07/14 03:00:47 | 11/07/14 09:00:37 | 6.00 | 9.58 | 0.70 |
| 8111_8112 | 8101_8102 | 11/29/14 17:20:11 | 11/29/14 18:41:08 | 1.35 | 46.58 | 0.20 |
| 8111_8112 | 8101_8102 | 11/29/14 21:40:16 | 11/29/14 22:40:15 | 1.00 | 52.29 | -0.22 |
| 8111_8112 | 8101_8102 | 11/30/14 03:00:43 | 11/30/14 04:00:14 | 0.99 | 76.45 | 0.09 |
| 8111_8112 | 8101_8102 | 11/30/14 10:20:14 | 11/30/14 12:20:44 | 2.01 | 66.23 | 0.04 |
| 8111_8112 | 8101_8102 | 12/09/14 08:20:15 | 12/09/14 12:00:14 | 3.67 | 35.81 | 0.02 |
| 8111_8112 | 8101_8102 | 12/16/14 00:00:43 | 12/16/14 07:00:43 | 7.00 | 78.6 | 0.15 |
| 8111_8112 | 8403_8404 | 11/15/14 12:00:37 | 11/16/14 06:00:44 | 18.00 | 51.97 | 0.37 |
| 8111_8112 | 8403_8404 | 11/25/14 18:20:40 | 11/25/14 20:00:14 | 1.66 | 44.4 | 0.20 |
| 8111_8112 | 8403_8404 | 11/30/14 08:40:11 | 11/30/14 11:00:12 | 2.33 | 45.6 | 0.43 |
| 8111_8112 | 8403_8404 | 12/09/14 08:20:15 | 12/09/14 09:20:15 | 1.00 | 53.7 | 0.03 |
| 8111_8112 | 8403_8404 | 12/15/14 07:00:43 | 12/15/14 14:00:43 | 7.00 | 51.25 | 0.76 |
| 8807_8808 | 8687_8688 | 11/24/14 21:40:13 | 11/25/14 00:01:07 | 2.35 | 25.97 | 0.41 |
| 8807_8808 | 8805_8806 | 12/03/14 22:20:08 | 12/04/14 02:00:12 | 3.67 | 75.33 | -0.04 |
| 8807_8808 | 8805_8806 | 12/04/14 10:20:38 | 12/04/14 13:20:11 | 2.99 | 67.88 | -0.15 |
| 8111_8112 | 8101_8102 | 10/20/15 07:00:44 | 10/20/15 16:00:40 | 9.00 | 50.84 | 0.38 |
| 8111_8112 | 8101_8102 | 11/01/15 21:01:55 | 11/02/15 21:01:07 | 23.99 | 56.41 | 0.17 |
| 8111_8112 | 8101_8102 | 11/09/15 03:01:08 | 11/09/15 06:00:43 | 2.99 | 74.79 | 0.37 |
| 12233_12307 | 8497_8498 | 11/08/17 11:00:42 | 11/08/17 15:01:30 | 4.01 | 72.46 | -0.16 |
| 8159_8160 | 8335_8336 | 11/08/14 18:00:44 | 11/09/14 00:00:43 | 6.00 | 31.48 | 0.52 |
| 8159_8160 | 8335_8336 | 12/07/14 16:40:11 | 12/07/14 17:40:12 | 1.00 | 32.84 | 0.87 |
| 8159_8160 | 8227_8228 | 12/02/13 20:20:12 | 12/03/13 05:20:15 | 9.00 | 45.87 | 0.50 |
| 8159_8160 | 8227_8228 | 12/06/13 05:20:14 | 12/06/13 06:20:28 | 1.00 | 60.47 | 0.74 |

Table A1-2: Summary of male-female interaction events (MFIE) using the conservative method for event identification.

| Male ID | Female ID | Start | End | Duration (h) | Mean Distance (m) | Mean DI |
| --- | --- | --- | --- | --- | --- | --- |
| 13686_13732 | 8101_8102 | 11/11/16 01:00:43 | 11/11/16 05:00:12 | 3.99 | 72.03 | 0.55 |
| 8111_8112 | 8101_8102 | 11/07/16 14:00:09 | 11/07/16 16:00:10 | 2.00 | 59.7 | 0.50 |
| 8111_8112 | 8101_8102 | 11/08/16 01:00:58 | 11/09/16 05:00:10 | 27.99 | 17.76 | 0.50 |
| 8111_8112 | 8101_8102 | 11/09/16 11:00:44 | 11/10/16 02:00:24 | 14.99 | 15.76 | 0.56 |
| 8111_8112 | 8101_8102 | 11/10/16 06:00:43 | 11/11/16 08:00:09 | 25.99 | 21.71 | 0.59 |
| 8111_8112 | 8101_8102 | 11/04/14 09:00:43 | 11/04/14 12:00:38 | 3.00 | 9.59 | 0.57 |
| 8111_8112 | 8101_8102 | 11/07/14 03:00:47 | 11/07/14 09:00:37 | 6.00 | 9.58 | 0.70 |
| 8111_8112 | 8403_8404 | 11/15/14 12:00:37 | 11/15/14 21:00:43 | 9.00 | 44.88 | 0.70 |
| 8111_8112 | 8403_8404 | 11/30/14 09:00:11 | 11/30/14 10:40:12 | 1.67 | 41.56 | 0.60 |
| 8111_8112 | 8403_8404 | 12/15/14 07:00:43 | 12/15/14 14:00:43 | 7.00 | 51.25 | 0.76 |
| 8807_8808 | 8687_8688 | 11/24/14 21:40:13 | 11/24/14 23:40:14 | 2.00 | 24.19 | 0.51 |
| 8111_8112 | 8101_8102 | 10/20/15 07:00:44 | 10/20/15 10:00:42 | 3.00 | 27.76 | 0.57 |
| 8111_8112 | 8403_8404 | 11/08/15 03:00:42 | 11/08/15 06:00:48 | 3.00 | 44.35 | 0.61 |
| 8159_8160 | 8335_8336 | 10/25/14 13:00:43 | 10/25/14 16:00:43 | 3.00 | 89.69 | 0.50 |
| 8159_8160 | 8335_8336 | 11/08/14 18:00:44 | 11/09/14 00:00:43 | 6.00 | 31.48 | 0.52 |
| 8159_8160 | 8335_8336 | 12/07/14 16:40:11 | 12/07/14 17:40:12 | 1.00 | 32.84 | 0.87 |
| 8159_8160 | 8227_8228 | 12/02/13 21:00:44 | 12/03/13 05:20:15 | 8.33 | 47.36 | 0.59 |
| 8159_8160 | 8227_8228 | 12/06/13 05:20:14 | 12/06/13 06:20:28 | 1.00 | 60.47 | 0.74 |

Table A1-3: Average UD volume during male-female interaction events (MFIE) using the liberal method for event identification.

| Event | Year | Male ID | Male $\bar{\mathrm{UD}}$ | Female ID | Female $\bar{\mathrm{UD}}$ |
| --- | --- | --- | --- | --- | --- |
| 1 | 2016 | 13686_13732 | 45.2 | 8101_8102 | 67.5 |
| 2 | 2016 | 8111_8112 | 39.7 | 8101_8102 | 17.5 |
| 3 | 2016 | 8111_8112 | 38.8 | 8101_8102 | 75.7 |
| 4 | 2014 | 8111_8112 | 75.8 | 8101_8102 | 49.1 |
| 5 | 2014 | 8111_8112 | 63.9 | 8101_8102 | 37.1 |
| 6 | 2014 | 8111_8112 | 14.8 | 8101_8102 | 45.9 |
| 7 | 2014 | 8111_8112 | 18.6 | 8101_8102 | 34.5 |
| 8 | 2014 | 8111_8112 | 15.2 | 8101_8102 | 44.9 |
| 9 | 2014 | 8111_8112 | 71.2 | 8101_8102 | 43.7 |
| 10 | 2014 | 8111_8112 | 29.8 | 8101_8102 | 29.6 |
| 11 | 2014 | 8111_8112 | 15.6 | 8101_8102 | 55.1 |
| 12 | 2014 | 8111_8112 | 46.7 | 8403_8404 | 39.8 |
| 13 | 2014 | 8111_8112 | 24.1 | 8403_8404 | 44.5 |
| 14 | 2014 | 8111_8112 | 65.9 | 8403_8404 | 40.5 |
| 15 | 2014 | 8111_8112 | 33.2 | 8403_8404 | 27.5 |
| 16 | 2014 | 8111_8112 | 34.3 | 8403_8404 | 35.1 |
| 17 | 2014 | 8807_8808 | 65.8 | 8687_8688 | 26.1 |
| 18 | 2014 | 8807_8808 | 10.7 | 8805_8806 | 36.7 |
| 19 | 2014 | 8807_8808 | 25.2 | 8805_8806 | 14.4 |
| 20 | 2015 | 8111_8112 | 9.5 | 8101_8102 | 38.0 |
| 21 | 2015 | 8111_8112 | 21.6 | 8101_8102 | 39.3 |
| 22 | 2015 | 8111_8112 | 31.4 | 8101_8102 | 1.8 |
| 23 | 2017 | 12233_12307 | 67.5 | 8497_8498 | 72.9 |
| 24 | 2014 | 8159_8160 | 20.6 | 8335_8336 | 66.2 |
| 25 | 2014 | 8159_8160 | 29.8 | 8335_8336 | 35.7 |
| 26 | 2013 | 8159_8160 | 12.0 | 8227_8228 | 25.4 |
| 27 | 2013 | 8159_8160 | 17.9 | 8227_8228 | 62.0 |

Table A1-4: Average UD volume during male-female interaction events (MFIE) using the conservative method for event identification.

| Event | Year | Male Individual | Male $\bar{\mathrm{UD}}$ | Female Individual | Female $\bar{\mathrm{UD}}$ |
| --- | --- | --- | --- | --- | --- |
| 1 | 2016 | 13686_13732 | 44.7 | 8101_8102 | 78.4 |
| 2 | 2016 | 8111_8112 | 36.6 | 8101_8102 | 14.2 |
| 3 | 2016 | 8111_8112 | 33.6 | 8101_8102 | 73.0 |
| 4 | 2016 | 8111_8112 | 46.1 | 8101_8102 | 84.0 |
| 5 | 2016 | 8111_8112 | 38.7 | 8101_8102 | 72.0 |
| 6 | 2014 | 8111_8112 | 75.8 | 8101_8102 | 49.1 |
| 7 | 2014 | 8111_8112 | 63.9 | 8101_8102 | 37.1 |
| 8 | 2014 | 8111_8112 | 52.1 | 8403_8404 | 51.7 |
| 9 | 2014 | 8111_8112 | 73.2 | 8403_8404 | 33.7 |
| 10 | 2014 | 8111_8112 | 34.3 | 8403_8404 | 35.1 |
| 11 | 2014 | 8807_8808 | 63.4 | 8687_8688 | 25.1 |
| 12 | 2015 | 8111_8112 | 11.0 | 8101_8102 | 46.9 |
| 13 | 2015 | 8111_8112 | 51.2 | 8403_8404 | 30.0 |
| 14 | 2014 | 8159_8160 | 13.2 | 8335_8336 | 47.7 |
| 15 | 2014 | 8159_8160 | 20.6 | 8335_8336 | 66.2 |
| 16 | 2014 | 8159_8160 | 29.8 | 8335_8336 | 35.7 |
| 17 | 2013 | 8159_8160 | 12.9 | 8227_8228 | 25.3 |
| 18 | 2013 | 8159_8160 | 17.9 | 8227_8228 | 62.0 |
