# Supplementary material for: Caution is warranted when using animal space-use and movement to infer behavioral states
Source: Mov Ecol. 2021 Jun 11;9:30. doi: 10.1186/s40462-021-00264-8 (PMC8196457; doi:10.1186/s40462-021-00264-8)

**Additional File 2**

Figure A2-1: Histogram of the UD volume (the smallest probability density contour that would contain the location) associated with white-tailed deer locations occurring during a male-female interaction event (MFIE) within the annual breeding season. MFIEs were identified using a liberal and conservative identification method for males (A and B respectively) and females (C and D respectively). The UD volume for each point was calculated as arising from a utilization distribution over the annual breeding season. The shading represents the duration of the MFIE associated with each location.


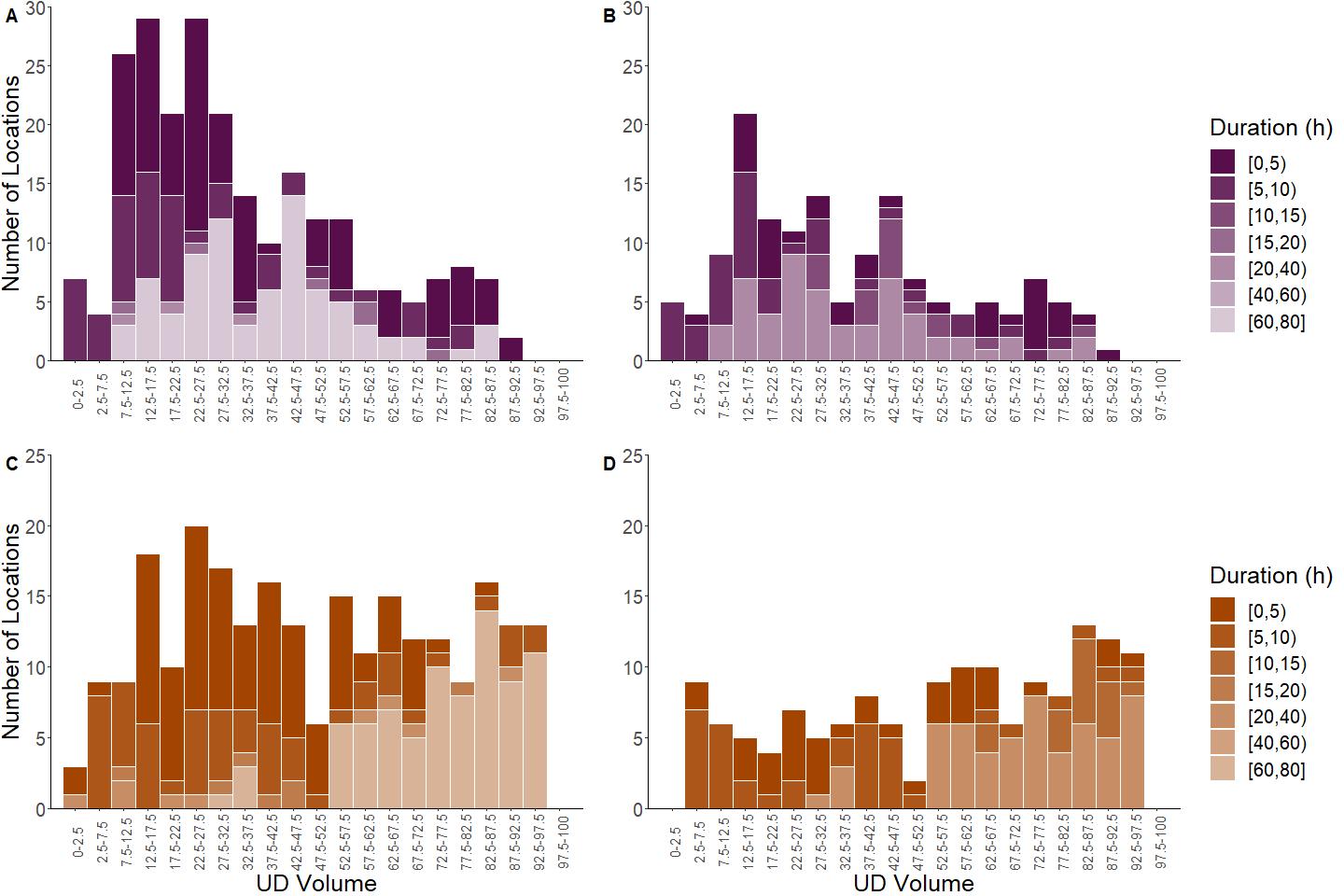


Figure A2-2: Proportions of male white-tailed deer locations identified as arising from two- (A and C) and three- state (B and D) HMMs during male-female interaction events (MFIEs) that were classified using the liberal event identification method (A and B respectively) and the conservative event identification method (C and D respectively) where HMMs were fit to telemetry data across the breeding season. Events are ordered from short to long MFIE durations.


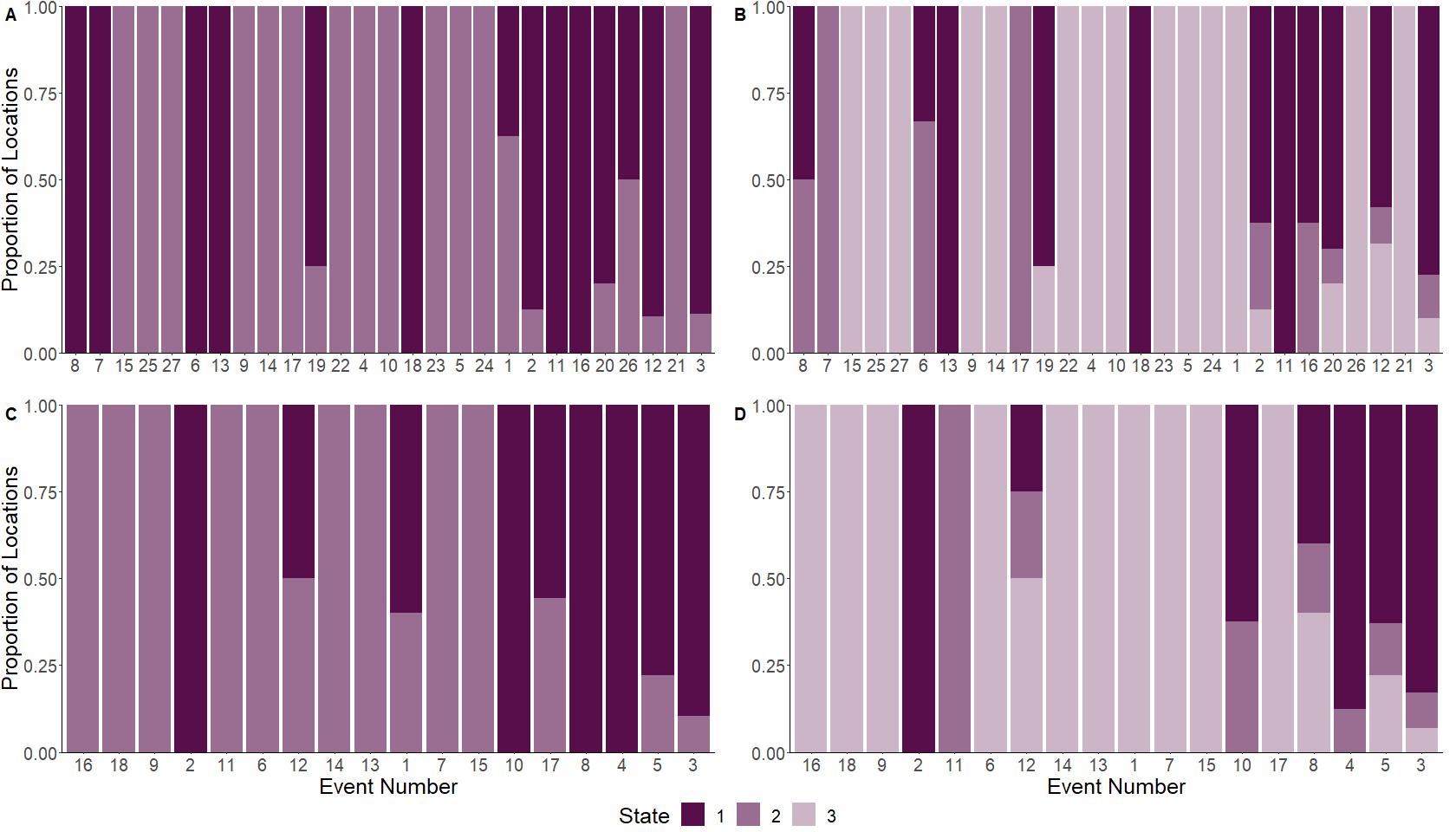


Figure A2-4: Proportions of male white-tailed deer locations identified as arising from a two-state HMM during male-female interaction events (MFIEs) that were classified from a liberal event identification method during early (A), peak (B), and late breeding phases (C) and a conservative event identification method during early (D), peak (E), and late (F) breeding phases where HMMs were fit to telemetry data across each phase of the breeding season. Events are ordered from small to large average distance between a male and female during the MFIE.


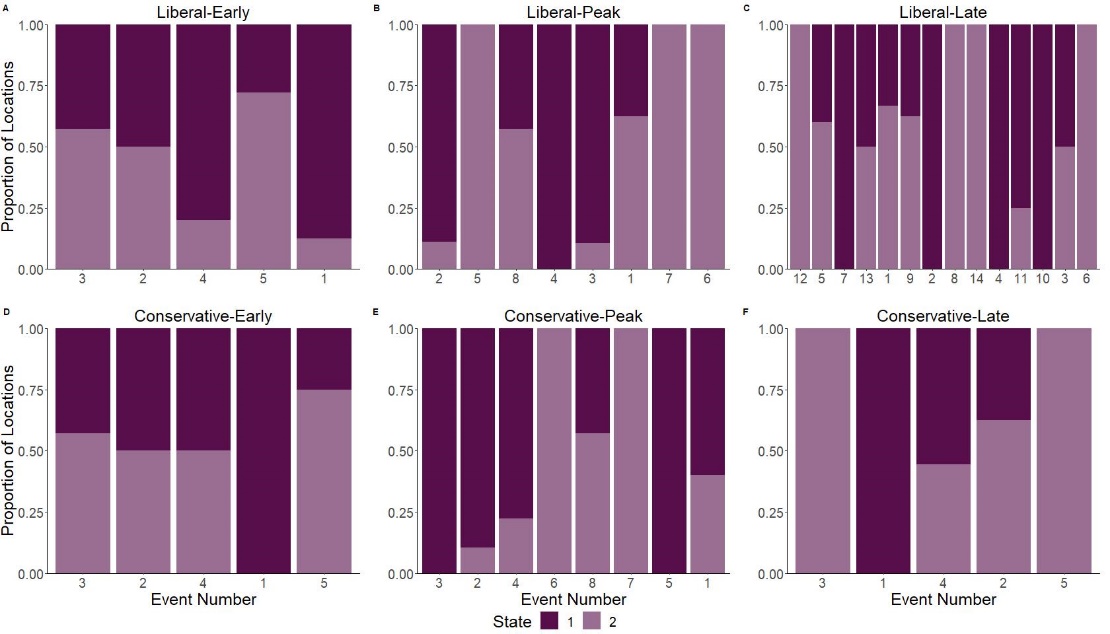


Figure A2-4: Proportions of male white-tailed deer locations identified as arising from a two-state HMM during male-female interaction events (MFIEs) that were classified from a liberal event identification method during early (A), peak (B), and late breeding phases (C) and a conservative event identification method during early (D), peak (E), and late (F) breeding phases where HMMs were fit to telemetry data across each phase of the breeding season. Events are ordered from short to long MFIE durations.


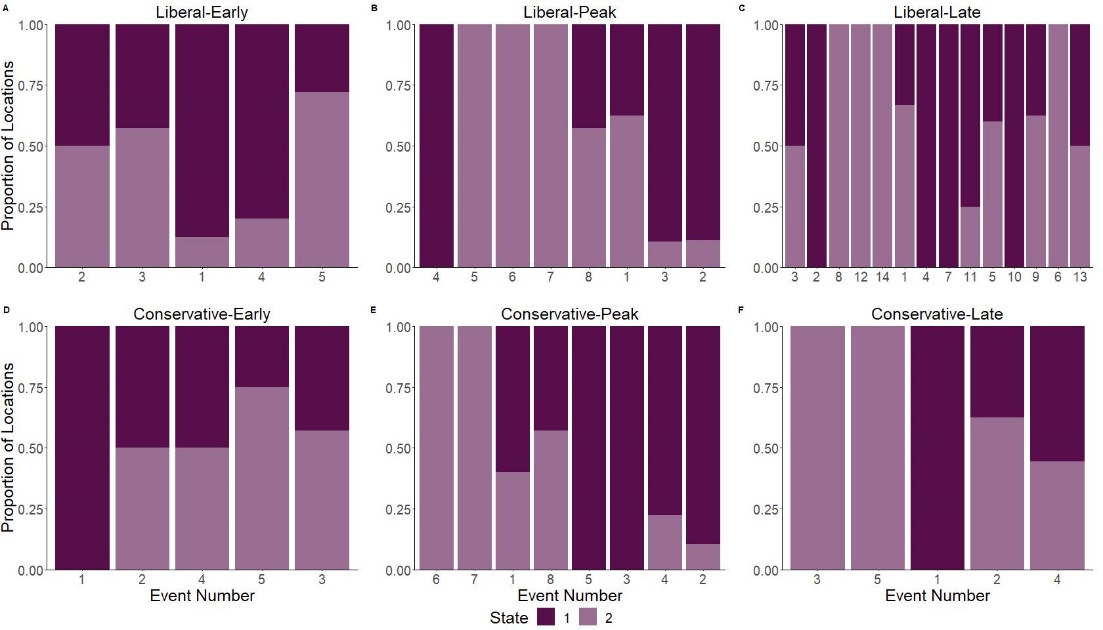


Figure A2-5: Proportions of male white-tailed deer locations identified as arising from a three-state HMM during male-female interaction events (MFIEs) that were classified from a liberal event identification method during early (A), peak (B), and late breeding phases (C) and a conservative event identification method during early (D), peak (E), and late (F) breeding phases where HMMs were fit to telemetry data across each phase of the breeding season. Events are ordered from small to large average distance between a male and female during the MFIE.


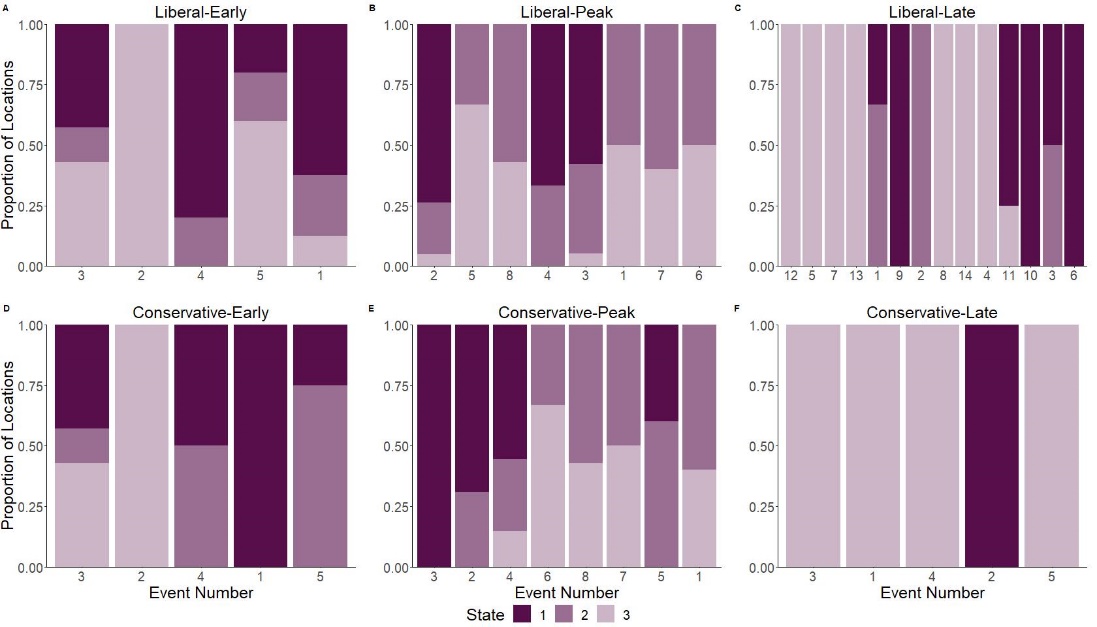


Figure A2-6: Proportions of male white-tailed deer locations identified as arising from a three-state HMM during male-female interaction events (MFIEs) that were classified from a liberal event identification method during early (A), peak (B), and late breeding phases (C) and a conservative event identification method during early (D), peak (E), and late (F) breeding phases where HMMs were fit to telemetry data across each phase of the breeding season. Events are ordered from short to long MFIE durations.


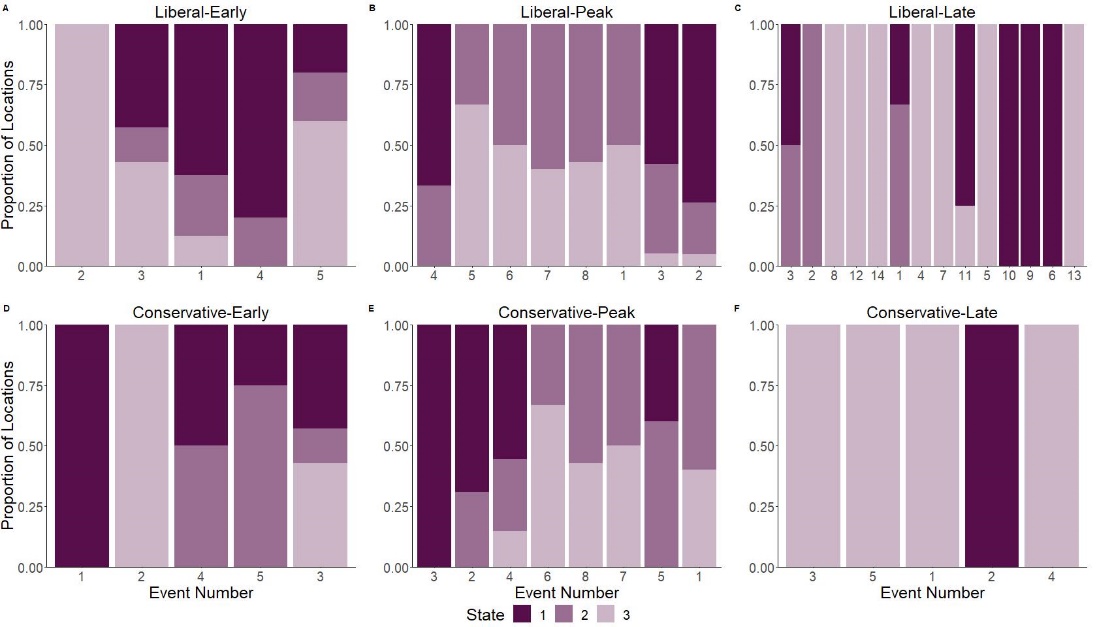

Supplement: Supplementary file 2 — Additional file 2. PDF including a visual representation of an example MFIE, expected results for the UD and HMM approach, and a summary of each male-female interaction event. [file 40462_2021_264_MOESM2_ESM.docx]
